# Fruit and Juice Epigenetic Signatures Are Associated with Independent Immunoregulatory Pathways

**DOI:** 10.3390/nu9070752

**Published:** 2017-07-14

**Authors:** Jessie Nicodemus-Johnson, Robert A. Sinnott

**Affiliations:** USANA Health Sciences, 3838 W Parkway Boulevard, West Valley City, UT 84120, USA; robert.sinnott@us.usana.com

**Keywords:** personalized nutrition, DNA methylation, epigenetics, fruit consumption, juice consumption

## Abstract

Epidemiological evidence strongly suggests that fruit consumption promotes many health benefits. Despite the general consensus that fruit and juice are nutritionally similar, epidemiological results for juice consumption are conflicting. Our objective was to use DNA methylation marks to characterize fruit and juice epigenetic signatures within PBMCs and identify shared and independent signatures associated with these groups. Genome-wide DNA methylation marks (Illumina Human Methylation 450k chip) for 2,148 individuals that participated in the Framingham Offspring exam 8 were analyzed for correlations between fruit or juice consumption using standard linear regression. CpG sites with low *P*-values (*P <* 0.01) were characterized using Gene Set Enrichment Analysis (GSEA), Ingenuity Pathway Analysis (IPA), and epigenetic Functional element Overlap analysis of the Results of Genome Wide Association Study Experiments (eFORGE). Fruit and juice-specific low *P*-value epigenetic signatures were largely independent. Genes near the fruit-specific epigenetic signature were enriched among pathways associated with antigen presentation and chromosome or telomere maintenance, while the juice-specific epigenetic signature was enriched for proinflammatory pathways. IPA and eFORGE analyses implicate fruit and juice-specific epigenetic signatures in the modulation of macrophage (fruit) and B or T cell (juice) activities. These data suggest a role for epigenetic regulation in fruit and juice-specific health benefits and demonstrate independent associations with distinct immune functions and cell types, suggesting that these groups may not confer the same health benefits. Identification of such differences between foods is the first step toward personalized nutrition and ultimately the improvement of human health and longevity.

## 1. Introduction

Fruit and vegetable consumption is a common dietary recommendation to support good health. Multiple components of fruits and vegetables (predominantly phytochemicals and fiber) have been shown to promote health and support immune function [1,2,3]. Moreover, epidemiological evidence has shown that increased fruit and vegetable consumption as part of a healthy diet reduces the incidence of a myriad of chronic inflammatory illnesses, such as cardiovascular diseases [4,5,6,7], cancer [8,9], asthma [10], and more generally mortality [11,12,13]. However, the results are conflicting for juice consumption [14,15,16,17,18] (reviewed in [19]). Currently, the USDA recommends a daily intake of 1.5–2 cups of fruit per day for healthy adults and reports that one-third of this daily intake of fruit is consumed in the form of juice [20]. Although juice is generally thought to be nutritionally similar to fruit, because they both contain polyphenols, vitamins, and minerals. In many instances, juice lacks the fiber component found in fruit which may alter the intestinal location and mechanism by which fruit derived nutrients are absorbed and ultimately processed [21] (reviewed in [22]). This difference could result in different health benefits conferred by each group. Given the broad role that fruit and juice consumption plays on immune function, such as inflammatory response and oxidative stress (reviewed in [2]), surprisingly little is known about the underlying molecular mechanisms by which these immunological health benefits are conferred. Knowledge of the mechanisms by which fruit or juice consumption modulate intrinsic cell signaling pathways and how these pathways relate to normal physiological function may contribute to improvement in human health and longevity, through personalization of nutritional intake. 

Epigenetics is a growing field that is capable of identifying underlying pathways associated with complex phenotypes. DNA methylation in particular is widely studied and has been shown to be a marker of environmental changes associated with disease (reviewed in [23]) and nutrition [3,24,25]. Identification of epigenetically-regulated pathways and molecules associated with these states have been instrumental in understanding the underlying molecular mechanisms associated with disease [26,27,28] or nutritional states [29,30] (reviewed in [31]). We hypothesized that the epigenetic signatures associated with fruit or juice intake will provide insight into the molecular mechanisms that underlie previously established physiological effects on immune function as well as allow assessment of the degree of shared and independent pathways between these two groups. To this end, we analyzed genome-wide DNA methylation profiles from 2,148 individuals of the Framingham Offspring cohort and discovered that fruit and juice consumption are largely comprised of independent epigenetic signatures (approximately 2% overlapping CpG sites) that target approximately 18% of the same genes. Pathway analyses demonstrate that genes near the fruit-associated epigenetic signature are enriched for immunosurveillance as well as DNA repair and maintenance pathways. While genes near the juice-associated epigenetic signature are enriched for proinflammatory signaling and immunotolerance pathways. Collectively, these data suggest that fruit and juice consumption associate with distinct areas of immune function, hinting that these foods may not confer the same health benefits.

## 2. Materials and Methods 

### 2.1. Study Participants

The present study included 2,148 Caucasian individuals that participated in the Framingham Heart Study Offspring cohort exam 8 from 2005–2008 and had all relevant phenotype information and genome-wide DNA methylation data available. Participants consisted of 979 men and 1,169 women ranging in age from 40–92 (median 65). As previously described [32], the FHS offspring cohort was recruited in 1971 and included 5124 offspring from the original FHS cohort and their spouses. Anthropomorphic measurements used were obtained at the Offspring exam 8.

### 2.2. Dietary Measures

Dietary intake was assessed with the semi quantitative Framingham food frequency questionnaire (FFQ) [33,34]. FFQs were mailed to non-institutionalized participants before the examination and the participants were asked to bring the completed questionnaire to their appointment. Participants reported how often, on average, they consumed a standard portion of each food item during the past year. Reported frequencies were used to estimate the number of usual daily/weekly servings of each item. Derived weekly servings of each food item were used in our analyses and used to create 2 categories: fruit and juice (Table S1). We used the residuals method to adjust the assessed foods for total energy intake. 

### 2.3. DNA Methylation Data Processing

DNA isolated from peripheral blood mononuclear cells (PBMCs) were assayed previously with the Infinium Human Methylation450K Bead Chip [35] (Illumina) and deposited in dbGaP [Study Accession: phs000724.v6.p10]. Genome-wide DNA methylation levels from 2,619 FHS participants were available for download. Probes located on the sex chromosomes or those that had detection *P*-values greater than 0.001 in 75% of samples were removed. Those mapping to more than one location in a bisulfite-converted genome or overlapping with the location of known single nucleotide polymorphisms (SNPs) were also removed [36]. Among the 485,000 probes on the array, 325,963 probes were carried forward and processed using the minfi package [37]. Infinium type I and type II probe bias was corrected for using the subset-quantile within array normalization (SWAN) algorithm [38]. Raw probe values were corrected for color imbalance and background by controls normalization. Methylation levels are reported as β values, which are the fraction of signal obtained from the methylated beads over the sum of methylated and unmethylated bead signals. Among the 2619 individuals available for analysis, 12 individuals failed QC and were removed from further analysis. 

Principal component analysis was used to determine the effects of known confounding variables on global methylation profiles. Chip, chip location, gender, age, and family relatedness were significantly associated with principal components (Figure S1, Table S2). Family relatedness was determined based on shared pedigrees, which included unrelated spouses as a conservative approach to account for shared environmental factors [39]. Chip and chip location effects were removed using COMBAT, while protecting fruit and juice consumption [40]. Surrogate variable analysis (SVA) was used to identify additional unknown technical or biological confounding variables in the COMBAT-adjusted residuals [41], such as white blood cell count composition [42]. Surrogate variables were also strongly associated with known covariates such as age, gender, relatedness, and disease status (Table S3). Residual COMBAT-adjusted methylation β values were used for all subsequent analyses. 

### 2.4. Statistical Analyses

Data were analyzed using R software (v3.3.1, R Foundation for Staistical Computing, Vienna, Austria). To assess the association of fruit consumption on DNA methylation levels at each CpG site, we performed a linear regression analysis using the R package limma [43] with the surrogate variables included as covariates. Gender, phenotype, relatedness, and disease status were captured by SVA (Table S3), and thus were not specifically accounted for in the regression model. Linear regression was performed on the 2,148 Caucasian individuals passing QC for which we had necessary phenotype, FFQ, and DNA methylation information. 

Empirical P-values were derived from 10,000 permutations. To assess low *P*-value enrichment, we permuted fruit or juice consumption then used linear regression to assess the correlation between CpG sites with a *P <* 0.01 (low *P*-value CpG sites; *N =* 5,221 and 5,434 for fruit and juice, respectively). Surrogate variables were again included as covariates in this model. We compared the *P*-value distributions between permuted and observed data using a *t*-test and recorded the number of times the permuted distribution was significantly elevated relative to the observed distribution. Empirical *P*-values for enrichment of overlapping low *P*-value CpG sites or nearby genes were determined by randomly selecting 5,221 and 5,434 CpG sites or 4,323 and 4,539 unique genes for fruit or juice consumption and recording the number of times the permuted overlap was greater than the observed overlap of 108 CpG sites or 1,246 genes. Violin plots and Venn diagrams were created using the R package vioplot and VennDiagram. DNase hypersensitivity site (DHS) enrichment for fruit and juice-specific epigenetic signatures were performed using epigenetic Functional element Overlap analysis of the Results of Genome Wide Association Study Experiments (eFORGE) [44]. eFORGE only accepts 1000 CpG sites, therefore a threshold of *P <* 0.001 was used for this analysis, resulting in 739 and 749 CpG sites submitted for fruit and juice analyses respectively.

### 2.5. Pathway Enrichment Analyses

Pathway enrichment analyses were performed using gene set enrichment analysis (GSEA) [45]. Genes within 5 kb of a low *P*-value CpG site were used. The top 100 pathways or those with a false discovery rate (FDR) <5% were reported. Gene lists of interest were also interrogated using Ingenuity Pathway Analysis (IPA) and network associations were constructed using the Ingenuity Knowledge Base. Network interactions were limited to those known in primary immune cells, while all other factors were kept at their default settings. The network score is based on the hypergeometric distribution of the network and is calculated with a right-tailed Fisher exact test to identify enrichment of those genes that were associated with fruit or juice consumption relative to the IPA database. A diagram illustrating the data analysis overview is presented in Figure S2. 

## 3. Results

### 3.1. Fruit and Juice Epigenetic Signatures 

**Figure 1:**
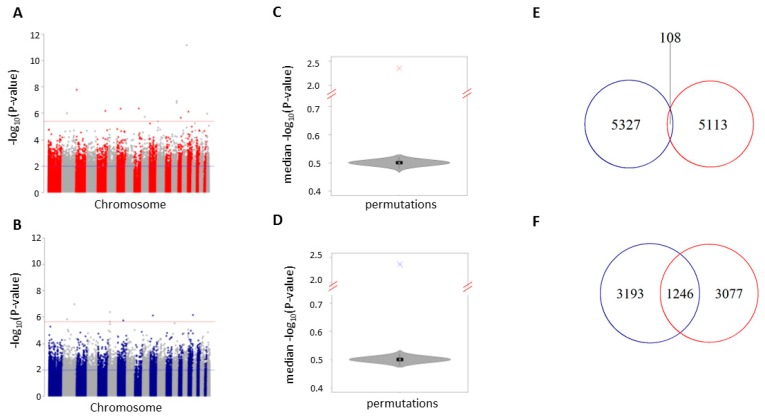
Fruit and juice epigenetic signatures. Manhattan plot of the 325,963 CpG sites in our analysis of fruit (**A**) or juice (**B**) -associated DNA methylation. The y-axis is the −log10 *P*-value of our regression analysis. −Log 10 *P*-values (y-axis) correspond to degree of correlation between DNA methylation and fruit consumption. The red line corresponds to a false discovery rate (FDR) threshold of 10%. The blue line corresponds to low *P*-value CpG sites (*P <* 0.01). Violin plot showing the distribution of median permutation *P*-values (*N =* 10,000) for the 5,221 and 5,434 shared CpG sites from fruit (**C**) and juice (**D**) analyses. Venn diagram depicting the number of shared and independent CpG sites (**E**) or genes (**F**) between fruit (red line) and juice (blue line) analyses.

The global effect of methylation changes on epigenetically regulated pathways of complex phenotypes can be ascertained from the combination of multiple CpG sites with relatively small effects and not the effects of individual CpG sites [26,27], thus we tested the P-value distribution of each study for an enrichment of low *P*-values (*P <* 0.01). There were 5,221 and 5,434 low *P*-value CpG sites for fruit and juice respectively (blue line; Figure 1A,B; Tables S4 and S5). Both fruit and juice consumption were enriched for low *P*-values (empirical *P <* 1 × 10^−5^ for both studies; Figure 1C,D; Figure S3). The epigenetic signatures (*P <* 0.01) associated with fruit or juice consumption were largely independent, with only two percent shared (108 CpG sites) between analyses. This is slightly more than expected by chance (empirical *P =* 0.01; expected overlap = 84). 

To assess whether low *P*-value CpG sites were near the same genes in each study, sites were mapped to the human genome (hg19 coordinates) and the gene with the nearest transcription start site was recorded. Among low *P*-value CpG sites that were within 5 kb of a gene transcription start site in each study, 27% (juice) and 29% (fruit) of associated genes overlapped or were shared between analyses (*N =* 1,246). This is enriched by 1.5-fold over the expected number of 804 shared genes (empirical *P <* 1 × 10^−5^). This suggests that although fruit and juice epigenetic signatures are largely independent, they may be influencing a large portion of the same genes. 

### 3.2. GSEA of Fruit and Juice Epigenetic Signatures 

To determine the pathways enriched among shared genes, we performed two separate gene set enrichment analyses: (1) genes near CpG sites in which the correlation between CpG site methylation levels and food consumption were in the same direction for both groups (*N =* 635 genes), i.e., increased fruit and juice consumption are both associated with increased CpG site methylation levels or increased consumption is associated with decreased CpG site methylation levels and (2) genes near CpG sites in which the correlation between CpG site methylation levels and food consumption were in opposite directions (*N =* 611 genes), i.e., decreased fruit consumption is associated with increased CpG site methylation levels and increased juice consumption is associated with decreased CpG site methylation levels and vice versa. Gene sets from the first analysis were enriched for many pathways associated with extracellular matrix assembly and function which may promote tissue development and homeostasis, such as core matrisome, extracellular matrix organization, and collagens, among others (Table S6A). Gene sets from the second analysis, i.e., genes associated with opposing epigenetic signatures, were enriched for many pathways associated with cell cycle, telomerase regulation, and development, but also genes involved in the immune system, specifically the adaptive immune system and pathways such as antigen processing and cross presentation (Table S6B). This suggests that fruit and juice epigenetic signatures that are associated with the same genes may have opposing influences on cell cycle and immune system-related pathways. 

Pathways enriched for genes near fruit and juice epigenetic signatures were identified using GSEA (*N =* 1,843 and 1,872 genes, respectively). While both fruit and juice epigenetic signatures were near genes enriched for many of the same pathways; immune system, cytokine signaling, and cell cycle for example, the genes and associative cell signaling pathways underlying these enrichments are quite different and unique to each group. The juice-specific epigenetic signature was enriched for innate and adaptive immune system genes, more specifically *transforming growth factor (TGF)-β*, *vascular endothelial growth factor (VEGF), toll-like receptor (TLR)4* and *nuclear factor kappa-light chain enhancer of activated B cells (NFk-β)* signaling pathways, among others (Table S7). Juice-specific cytokine signaling pathway genes include *myeloid differentiation primary response (MYD)88*, *interferon regulatory factor* (*IRF)8* and *IRF4*, which are immune specific transcription factors required for immune cell processes such as T cell differentiation to T helper (Th)2 and Th17 or activation of B cells (reviewed in [46]). Juice consumption has been broadly associated with enhanced immune function; these data suggest specific epigenetically regulated proinflammatory pathways that may contribute.

In contrast, immune system genes that are associated with the fruit-specific epigenetic signature were enriched for only adaptive immune system pathways, specifically antigen processing presentation. Cytokine signaling genes near the fruit-specific epigenetic signature include *human leukocyte antigen (HLA)-F* and *HLA-DPB1*, both molecules involved in antigen presentation and immune cell activation. This is consistent with a previous interventional study in elderly individuals, where fruit consumption was associated with increased antigen presentation [47]. Moreover, both groups were enriched for general cell cycle, meiosis and mitosis pathways, but the fruit-specific epigenetic signature was associated with 17% more genes in these pathways than the juice-specific epigenetic signature (75 versus 62 genes, respectively). Additional fruit-specific pathways include those involved in cell cycle regulation and chromosome or telomere maintenance, which are important to promote healthy growth and aging of the immune system (Tables S7 and S8) [48,49]. Collectively, these data indicate fruit and juice consumption-associated epigenetic modifications may influence different areas of immune system function.

### 3.3. IPA of Fruit and Juice Epigenetic Signatures Near Shared Genes

**Figure 2:**
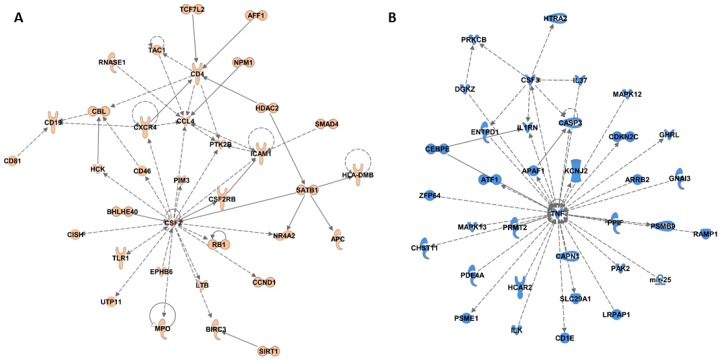
Ingenuity pathway analysis protein–protein interaction networks derived from genes within 5 kb of a low P-value CpG site (*P <* 0.01). (**A**) Fruit associated network 1 is centered on colony stimulating factor (CSF)2, cluster of differentiation (CD)4, and C-C motif chemokine ligand (CCL)4 (network score 38); (**B**) Juice associated network 1 is centered on tumor necrosis factor (TNF) and colony stimulating factor (CSF)3 (network score 38). Genes that were associated with fruit or juice-specific epigenetic signatures are colored in orange or blue, respectively.

DNA methylation profiles of WBCs are derived from a pool of lymphocytes. To elucidate epigenetically associated pathways that may implicate specific lymphocyte populations, we performed IPA network analyses to identify specific protein–protein interaction networks that are enriched for genes near fruit and juice-specific epigenetic signatures. The fruit-specific analysis resulted in two significant protein–protein interaction networks (Figure 2A; Figure S4A; network scores 38 for both). One network is centered around colony stimulating factor (CSF)2, chemokine ligand (CCL)4, and cluster of differentiation (CD)4 (Figure 2A), all molecules associated with macrophage attraction, proliferation, and activation [50,51]. This is consistent with the above GSEA enrichment of antigen presenting processes and suggests that macrophage mediated antigen presentation may be influenced by fruit-specific epigenetic signatures. 

The juice-specific analysis also produced two significant networks (Figure 2B; Figure S4B; network score 38 for both). The first network (Figure 2B) is centered on tumor necrosis factor (TNF)α, a major proinflammatory cytokine that mediates innate immune system acute inflammatory responses, and CSF3, a molecule that stimulates granulocyte production in bone marrow and release into the bloodstream [52]. The second is centered on MYD88, IRF8, IRF4 and inhibitor of DNA binding (ID)3, immune specific transcription factors required for immune cell processes such as T cell differentiation to Th2 and Th17 or activation of B cells [53] (reviewed in [46]), as well as C-C motif chemokine receptor (CCR)7 a molecule associated with Th1 cell differentiation and tolerance [54]. These data concur with GSEA analyses that implicated innate and adaptive immune pathway association with the juice epigenetic signature. Collectively, IPA analyses add further information on fruit or juice-specific epigenetically associated cell signaling pathways by which immunotolerance (fruit) or inflammatory processes (juice) may be influenced. 

### 3.4. DHS Enrichment Analysis 

To increase our knowledge of the immune cell populations that are associated with juice and fruit-specific epigenetic signatures, we scanned genome-wide DNase hypersensitivity sites from specific immune cell populations for enrichment of these signatures using the online program eFORGE [44]. The fruit-specific epigenetic signature (*N =* 739 CpG sites; *P <* 0.001) was enriched within natural killer (NK) cell DHS (*P =* 0.00011; Figure S5A). The juice-specific epigenetic signature (*N =* 749 CpG sites; *P <* 0.001) was enriched for primary peripheral blood T, NK, and B cell DHS, as well as primary monocyte DHS (*P =* 5.14 × 10^−6^, 9.17 × 10^−6^, 0.0018, 4.65 × 10^‒4^ respectively; Figure S5B). This is consistent with IPA analyses, which highlight the enrichment of cell signaling pathways associated with B and T cell differentiation and activation. To assess the direction of effect, i.e., whether increased juice consumption was correlated with reduced CpG site methylation levels (negatively correlated) or with increased methylation levels (positively correlated), we stratified the CpG sites with a *P <* 0.001 by those that were positively or negatively correlated with fruit or juice consumption and re-ran the eFORGE analyses. There was not an enrichment for either subset of fruit-specific CpG sites or positively correlated juice CpG sites (Figure S5C–E). Negatively correlated CpG sites were enriched for primary T (*P =* 2.22 × 10^−4^) and NK (*P =* 5.63 × 10^−5^) cell DHS (Figure S5F), suggesting that increased juice consumption may reduce global DNA methylation levels within DHS of specific primary immune cell populations, specifically T and NK cell regulatory regions. 

To better understand what pathways the above sites may influence in B and T cells, we performed IPA protein–protein interaction network analysis to identify specific pathways enriched for genes near low *P*-value CpG sites that had less methylation with increased juice consumption. This analysis produced two networks. One network was centered on TNF (Figure S6; network score = 43) a major proinflammatory molecule, while the second was centered on ID3, protein tyrosine phosphatase, non-receptor type 6 (PTPN6), and CCR7 (network score = 43), molecules involved in hematopoietic cell differentiation [55] and tolerance [54,56]. Reduced methylation in promoter regions is frequently associated with increased gene expression, therefore the observed reduction in promoter methylation with increased juice intake may enhance pathway signaling upon NK or T cell activation. Interestingly, increased NK cell lytic activity has been associated with increased fruit juice consumption [57]. Collectively, this suggests that juice-specific epigenetic signatures may promote enhanced immune responses among activated NK and T cells, a subset of which also supports increased cell differentiation and immunotolerance. 

## 4. Discussion

The field of nutritional epigenomics allows the molecular level assessment of nutrient-induced changes in the body through the identification of nutrient-gene or more globally nutrient–pathway interactions. Nutrient-induced epigenetic modifications can alter a myriad of cellular responses to environmental stimuli [58], such as immune response to infection. We applied this approach to compare and contrast the epigenetically associated pathways correlated with fruit or juice consumption in PBMCs. In general, our findings support the well-defined effect of fruit and juice consumption on immune health, specifically reduced DNA damage and immune system activation (reviewed in [2,59]). Moreover, we demonstrate that fruit and juice-associated epigenetic signatures are distinct from one another and associated with different underlying cell signaling pathways. This was observed not only in independent fruit and juice-specific epigenetic signatures but also among shared genes which appeared to be associated with opposing epigenetic signatures (and presumably gene expression) among immune related pathways. This is in contrast to the common conception that fruit and juice are nutritionally similar and thus confer similar beneficial effects. In fact, the data presented suggest that fruit and juice consumption modulate different aspects of immune function, with genes near the juice epigenetic signature enriched for pathways associated with proinflammatory response and immunotolerance, while genes near the fruit epigenetic signature are enriched for immunosurveillance and chromosome or telomere maintenance pathways. Collectively, our results suggest that fruit and juice consumption may not confer the same immune health benefits and provides novel pathways and immune functions for further study.

The epigenetic differences observed in our study may be attributable to variation in fruit fiber content between fruit and juice. Fiber, a largely indigestible molecule, alters the digestion rate of co-consumed nutrients and thus influences the intestinal location and mechanism by which fruit derived nutrients are absorbed and ultimately processed (reviewed in [22]). Much of this variation in intestinal absorption is likely due to variation in breakdown of nutrients by intestinal microbiota [21,60,61], which vary in composition throughout the intestine. In support of this, an observational study demonstrated that the anti-inflammatory effects of fruit and vegetable consumption were higher in individuals with elevated fiber intake from fruit and vegetables [62]. This suggests that individuals who consume more juice may benefit from ingestion of additional forms of fruit fiber. Regardless of the cause, our observational findings warrant further study into the specific immunological benefits of fruit and juice consumption. 

We note limitations to our study. Due to the nature of observational studies, our findings are correlative and cannot infer causality. Additionally, we cannot discount that a portion of our findings may be due to additional foods that may be routinely co-consumed with fruit or juice. However, based on the literature support for our conclusions, we believe this is unlikely.

We demonstrate for the first time that juice and fruit consumption are correlated with global epigenetic variation and that these largely independent signatures suggest that fruit and juice consumption influence different immune cell populations and different aspects of immune function, specifically immunosurveillance and proinflammatory pathway activation respectively. Additionally, our analyses implicate novel epigenetically regulated target molecules and pathways associated with these groups that afford new insight into the underlying molecular mechanisms of these associations. An understanding of how nutritional intake contributes to physiological phenotypes, such as immune function, is the first step toward utilization of nutrition to improve human health and ultimately personalized nutrition. 

## Supplementary Materials:

The following are available online at www.mdpi.com/2072-6643/9/7/752/s1, Figure S1: Principal component analyisis of 2386 individuals with genome-wide DNA methylation data. Figure S2: Data analysis overview diagram. Figure S3: *P-*value distributions of fruit and juice linear regression analysis. Figure S4: Ingenuity pathway analysis protein–protein interaction networks derived from genes within 5 kb of a low *P*-value CpG site. Figure S5: eFORGE analysis of fruit- and juice-specific CpG sites (*P <* 0.001). Figure S6: Ingenuity pathway analysis protein–protein interaction networks derived from genes within 5 kb of a negatively correlated juice-specific low *P*-value CpG site (*P <* 0.01). Table S1: Definitions for fruit and vegetable groupings, Table S2: Principal Component Analysis Outputs, Table S3: Correlation between surrogate variables (columns) and available Framingham traits (rows), Table S4: Linear regression results for fruit consumption, Table S5: Linear regression results for juice consumption, Table S6: Gene set enrichment analysis results (FDR 5%) for shared genes. Table S7: Gene set enrichment analysis results for genes associated with the fruit-specific epigenetic signature. Table S8: Gene set enrichment analysis results for genes associated with the juice-specific epigenetic signature.
