# Correction: Nicodemus-Johnson, J.; et al. Fruit and Juice Epigenetic Signatures Are Associated with Independent Immunoregulatory Pathways. *Nutrients* 2017, *9*, 752

**DOI:** 10.3390/nu9091038

**Published:** 2017-09-20

**Authors:** Jessie Nicodemus-Johnson, Robert A. Sinnott

**Affiliations:** USANA Health Sciences, 3838 W Parkway Boulevard, West Valley City, UT 84120, USA; robert.sinnott@us.usana.com

We would like to submit the following correction to our recently published paper [1] due to the error in illustration of the abbreviation eFORGE. The details are as follows:

(1) The sentence in the abstract “and epigenetic Functional element Overlap analysis of the Results of Genome Wide Association Study Experiments (eFORGE).” has been changed to “and experimentally derived Functional element Overlap analysis of ReGions from EWAS (eFORGE).”
(2) In the last paragraph of Section 2.4, the sentence “DNase hypersensitivity site (DHS) enrichment for fruit and juice-specific epigenetic signatures were performed using epigenetic Functional element Overlap analysis of the Results of Genome Wide Association Study Experiments (eFORGE) [44].” has been changed to “DNase hypersensitivity site (DHS) enrichment for fruit and juice-specific epigenetic signatures were performed using experimentally derived Functional element Overlap analysis of ReGions from EWAS (eFORGE) [44].”

We apologize for any inconvenience caused to our readers.
