# Supplementary material for: Fruit and Juice Epigenetic Signatures Are Associated with Independent Immunoregulatory Pathways
Source: Nutrients. 2017 Jul 14;9(7):752. doi: 10.3390/nu9070752 (PMC5537866; doi:10.3390/nu9070752)
Supplement: Supplementary file 1 [file nutrients-09-00752-s001.zip › Supplementary Materials_final2.pdf]

Supplementary Materials

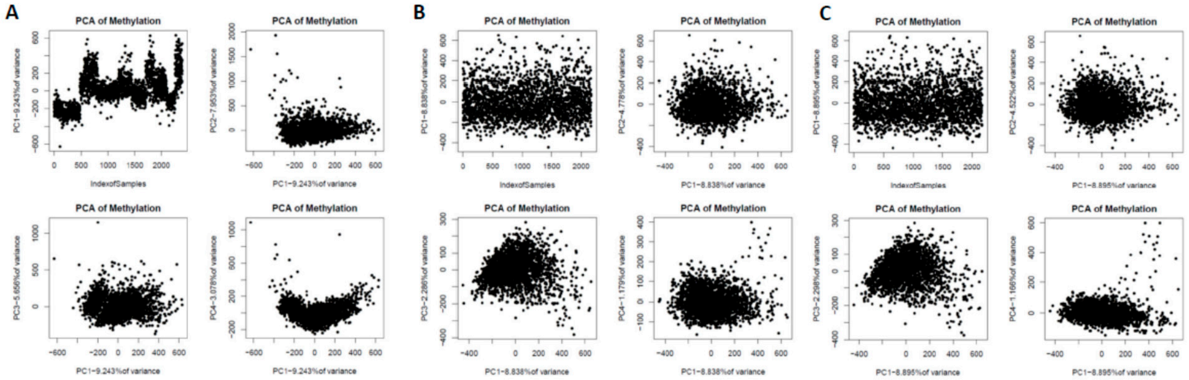

Figure S1. Principal component analysis (PCA) of 2,386 individuals with genome-wide DNA methylation data. Scatter plot of Principal component (PC) 1 (top left panel), PC1 relative to PC2 (top right panel), PC1 relative to PC3 (bottom left panel), and PC1 relative to PC4 (bottom right panel). PC scatter plots were generated from PCA after data QC (A), after removal of chip effect (B), and after removal of chip location effect (C). Axes are labeled with the PC followed by the proportion of variance explained by that PC.

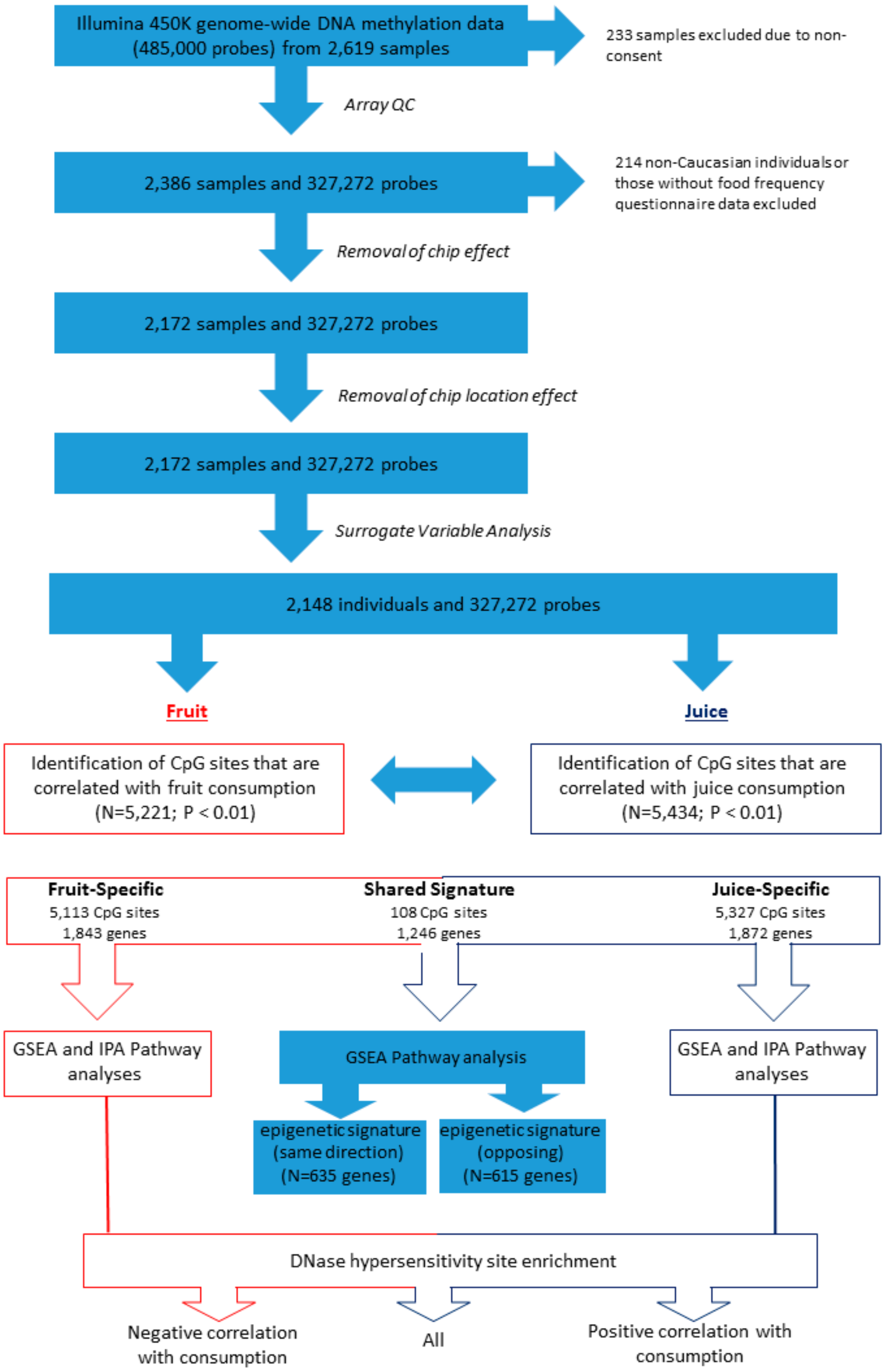

Figure S2. Data analysis overview diagram. Blue filled boxes represent processes performed on both fruit and juice epigenetic data. Red outline boxes are analyses performed separately for fruit. Blue outline boxes are analyses performed separately for juice.

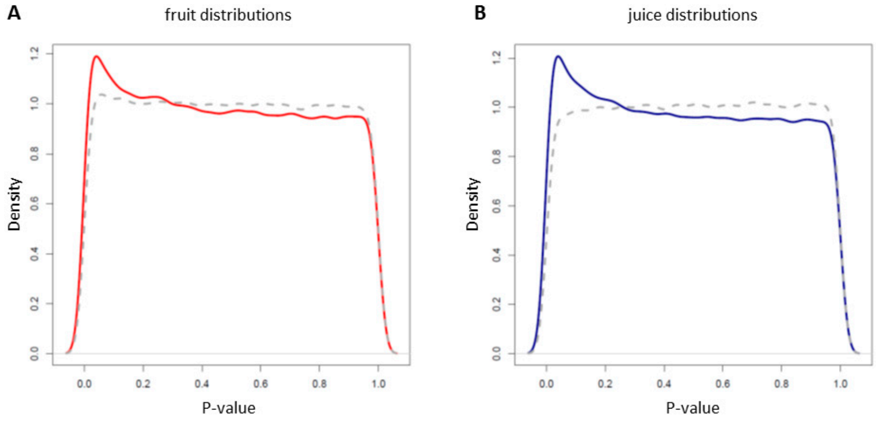

Figure S3. P-value distributions of fruit and juice linear regression analysis. Density plot of CpG site P-values for fruit **(A)** and juice **(B)** analyses. Fruit or juice-specific distributions are shown in red and blue respectively. The distribution from one permutation is shown as a dotted grey line.

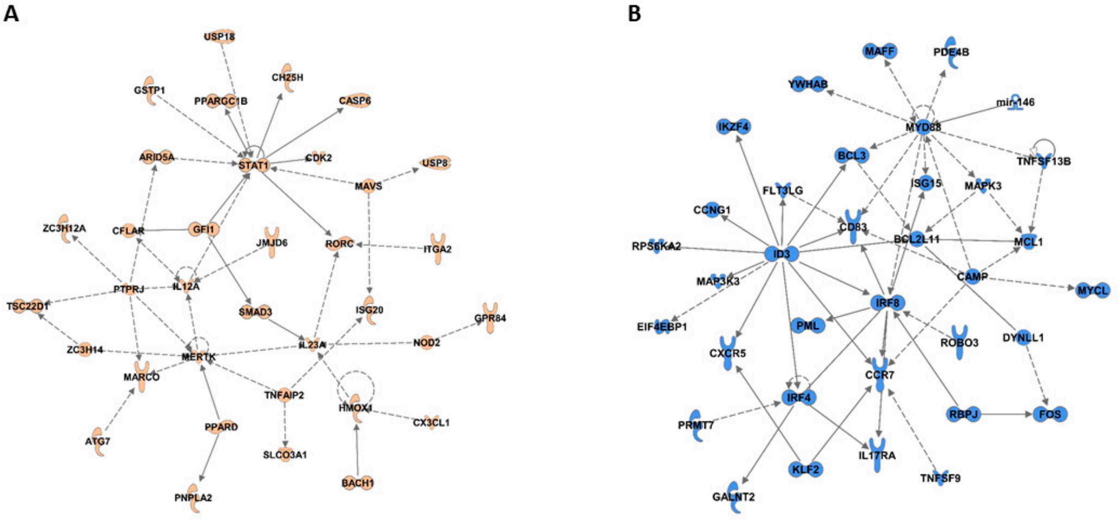

Figure S4. Ingenuity pathway analysis protein-protein interaction networks derived from genes within 5 kb of a low P-value CpG site. **(A)** Fruit-associated network 2 (network score 38). **(B)** Juice-associated network 2 (network score 38).

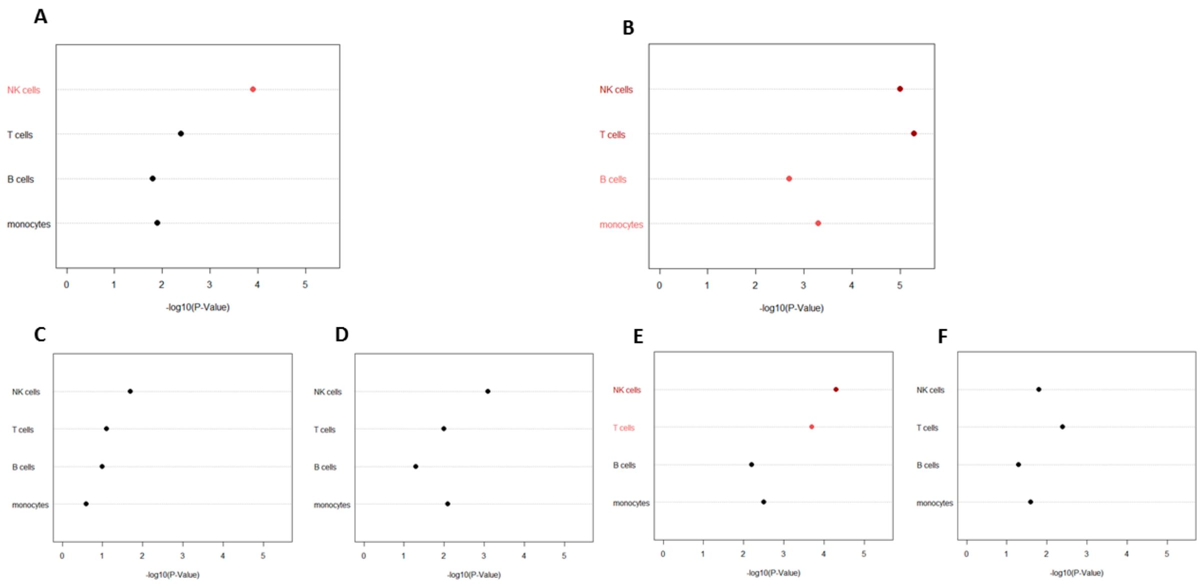

Figure S5. Fruit- and juice-specific epigenetic signature enrichment for immune cell DHS. –log10 enrichment P-values (x-axis) for different immune cell populations (y-axis) are shown. eFORGE analyses of **(A)** all fruit-specific CpG sites ($P < 0.001$), **(B)** all juice- specific CpG sites, CpG sites ($P < 0.001$) that are negatively **(C)** and positively **(D)** correlated with fruit consumption, or negatively **(E)** and positively **(F)** correlated with juice consumption.

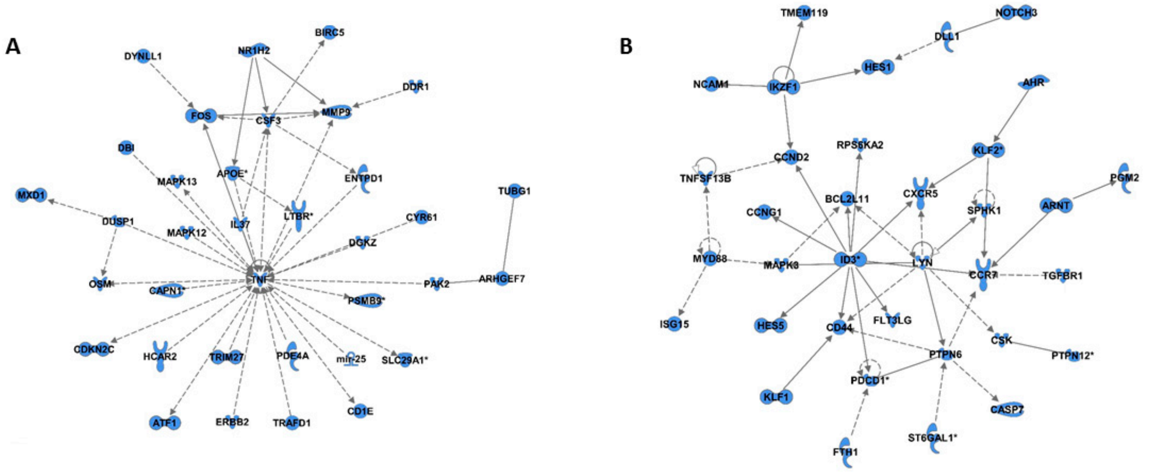

Figure S6. Ingenuity pathway analysis protein-protein interaction networks derived from genes within 5 kb of a negatively correlated juice-specific low P-value CpG site (P< 0.01). **(A)** Fruit associated network 1 is centered on TNF (network score 43) and **(B)** network 2 is centered on ID3 and PTPN6 (network score 43).
